# The Brief Negative Symptom Scale: external validation of symptom domains with clinical, cognitive and functioning-related variables in subjects with schizophrenia

**DOI:** 10.1192/j.eurpsy.2023.602

**Published:** 2023-07-19

**Authors:** G. M. Giordano, A. Mucci, P. Rucci, F. Sanmarchi, E. Caporusso, L. Giuliani, A. Perrottelli, P. Pezzella, P. Bucci, P. Rocca, A. Rossi, A. Bertolino, S. Galderisi, M. Maj

**Affiliations:** ^1^Department of Psychiatry, University of Campania “Luigi Vanvitelli”, Naples; ^2^Department of Biomedical and Neuromotor Sciences, University of Bologna, Bologna; ^3^Department of Neuroscience, Section of Psychiatry, University of Turin, Turin; ^4^Section of Psychiatry, Department of Biotechnological and Applied Clinical Sciences, University of L’Aquila, L’Aquila; ^5^Department of Basic Medical Science, Neuroscience and Sense Organs, University of Bari ‘Aldo Moro’, Bari, Italy

## Abstract

**Introduction:**

Negative symptoms (NS) represent a heterogeneous construct of schizophrenia, whose conceptualization is still to be clarified. In the last decade, the conceptualization model that has received the most support from the literature has described 2 NS domains: the expressive deficit (EXP), which includes blunted affect and alogia, and the motivational deficit (MAP), which includes avolition, asociality, and anhedonia. However, different confirmatory factor-analytic studies suggest that the bi-dimensional model may not capture the complexity of this construct, which could be better defined by a 5-factor model (5 individual negative symptoms) or a hierarchical model (5 individual negative symptoms as first-order factors, and the 2 domains, MAP and EXP domains, as second-order factors). However, to our knowledge, no study has investigated associations between negative symptom models with social cognition and functional capacity, which are largely documented to correlate with negative symptoms, nor the associations with external validators over time, looking at the potential stability of negative symptom models validity through the course of the illness.

**Objectives:**

In the light of this observations, we investigated, the external validity of the five-factor model and the hierarchical model of the BNSS in subjects with schizophrenia, looking at associations with cognition, social cognition, functioning and functional capacity at baseline and at four years follow-up.

**Methods:**

NS were assessed in 612 subjects with schizophrenia using the Brief Negative Symptom Scale at the baseline and after 4-year follow-up. State of the art assessment instruments were used to assess cognitive and functioning related variables. Structural equation models (SEM) that included the NS models and 4 external variables were used to our aim.

**Results:**

According to recent multicenter studies, our results confirmed the validity of the 5-factor- and the hierarchical-model of negative symptoms. In particular, these 2 models proved to be equivalent in terms of fit to the data at baseline and follow-up. As regard to the relationship of the two BNSS models with external variables, we found that there was a similar pattern of associations at the two time points despite minor variations.

**Conclusions:**

The five factor and the hierarchical models provide an optimal conceptualization of negative symptoms in relation to external variables. The similar pattern of associations with external variables of the two models at the two time points despite minor variations, suggests that the simple and widely used 5-factor solution provides the best balance between parsimony and granularity to summarize BNSS structure. This data is of important relevance with consequent implications in the study of pathophysiological mechanisms and the development of targeted treatments for NS.

**Disclosure of Interest:**

None Declared

